# Machine Learning-Based Characterization of the Nanostructure in a Combinatorial Co-Cr-Fe-Ni Compositionally Complex Alloy Film

**DOI:** 10.3390/nano12244407

**Published:** 2022-12-10

**Authors:** Péter Nagy, Bálint Kaszás, István Csabai, Zoltán Hegedűs, Johann Michler, László Pethö, Jenő Gubicza

**Affiliations:** 1 Department of Materials Physics, Eötvös Loránd University, 1117 Budapest, Hungary; 2 Laboratory for Mechanics of Materials and Nanostructures, Empa, Swiss Federal Laboratories for Materials Science and Technology, 3602 Thun, Switzerland; 3 Institute for Mechanical Systems, ETH Zürich, 8092 Zurich, Switzerland; 4 Department of Physics of Complex Systems, Eötvös Loránd University, 1117 Budapest, Hungary; 5 Deutsches Elektronen-Synchrotron DESY, 22607 Hamburg, Germany

**Keywords:** artificial intelligence, machine learning, X-ray line profile analysis, compositionally complex alloy, nanostructure

## Abstract

A novel artificial intelligence-assisted evaluation of the X-ray diffraction (XRD) peak profiles was elaborated for the characterization of the nanocrystallite microstructure in a combinatorial Co-Cr-Fe-Ni compositionally complex alloy (CCA) film. The layer was produced by a multiple beam sputtering physical vapor deposition (PVD) technique on a Si single crystal substrate with the diameter of about 10 cm. This new processing technique is able to produce combinatorial CCA films where the elemental concentrations vary in a wide range on the disk surface. The most important benefit of the combinatorial sample is that it can be used for the study of the correlation between the chemical composition and the microstructure on a single specimen. The microstructure can be characterized quickly in many points on the disk surface using synchrotron XRD. However, the evaluation of the diffraction patterns for the crystallite size and the density of lattice defects (e.g., dislocations and twin faults) using X-ray line profile analysis (XLPA) is not possible in a reasonable amount of time due to the large number (hundreds) of XRD patterns. In the present study, a machine learning-based X-ray line profile analysis (ML-XLPA) was developed and tested on the combinatorial Co-Cr-Fe-Ni film. The new method is able to produce maps of the characteristic parameters of the nanostructure (crystallite size, defect densities) on the disk surface very quickly. Since the novel technique was developed and tested only for face-centered cubic (FCC) structures, additional work is required for the extension of its applicability to other materials. Nevertheless, to the knowledge of the authors, this is the first ML-XLPA evaluation method in the literature, which can pave the way for further development of this methodology.

## 1. Introduction

Compositionally complex alloys (CCAs) are at the forefront of materials science since these materials represent the undiscovered middle parts of the multicomponent phase diagrams [1]. Therefore, CCAs may exhibit a unique combination of properties which may open the door for novel applications. CCAs contain at least three different constituents with equal or near-equal fractions [1,2]. Since, in this case, there are no solute and solvent elements in the alloy, these materials are also referred to as multi-principal element alloys (MPEAs). This class of materials includes high-entropy alloys (HEAs) where the minimum number of constituent elements is five, with atomic concentrations between 5 and 35% [3]. In the case of HEAs, a single phase state can be achieved due to the stabilization effect of the high entropy caused by the large number of constituents with comparable fractions [3]. The novel structures of CCAs can yield improved behavior compared to conventional alloys. For instance, a combination of high strength and good ductility can be achieved for some compositions [4]. Furthermore, refractory CCAs can exhibit superior strength values even at very high temperatures [5].

Due to the large variety of the selectable chemical elements, many different CCAs can be produced. In addition, for a selected group of constituents, their concentrations can also be varied, which may result in very different structures yielding a large variety of properties of CCAs [6]. If the constituent elements were selected, mapping the structure and properties of HEAs versus the atomic concentrations of the constituents would require manufacturing of a large number of samples. Rather, it is simpler to study the effect of the chemical composition on the structure and properties using combinatorial samples. Such samples have been produced in the form of films on reasonably large substrates using deposition techniques [6,7,8,9,10]. For instance, Fe-Mn-Co-Cr-Al, Co-Cr-Fe-Mn-Ni, Al-Co-Cr-Fe-Ni, Co-Cr-Fe-Ni and Ag-Ir-Pd-Pt-Ru systems were studied using combinatorial film samples processed with co-deposition methods, such as multiple-beam sputtering (MBS) [9]. The effect of composition on the structure and the properties (e.g., mechanical and magnetic) was revealed successfully.

Structure characterization on combinatorial samples in a reasonable time and with an appropriately fine resolution can be performed using synchrotron X-ray diffraction (XRD). In this case, the acquisition time of obtaining the XRD patterns is very low; however, their evaluation takes an extremely long time if the structural mapping is performed with a fine compositional resolution. For instance, in a former study for a CoCrFeNi CCA combinatorial film, the step size of XRD mapping was about 2–4 mm, which corresponded to a compositional resolution of about 1 at.% [11]. Taking the 10 cm diameter of the sample into account, thousands of XRD patterns should be evaluated for a complete structural mapping. If the XRD patterns are also evaluated for the diffraction peak profiles in order to determine the crystallite size and the defect density, a complete mapping using conventional profile fitting methods is not feasible in a reasonable time.

In this study, a novel method based on artificial intelligence is proposed for a fast evaluation of XRD patterns for the determination of the crystallite size and the density of crystal defects, such as dislocations and twin faults in face-centered cubic (FCC) structures. The new machine learning-based X-ray line profile analysis (ML-XLPA) technique is capable of making maps of the microstructural parameters for FCC combinatorial CCAs. The effectiveness of the new ML-XLPA method was demonstrated on a nanocrystalline Co-Cr-Fe-Ni CCA processed with MBS technique. The novel ML-XLPA is validated by comparing the obtained microstructural parameters with those determined using traditional pattern fitting method. It should be noted that machine learning has already been applied for the characterization of the crystal structure of materials based on diffraction. For instance, an artificial intelligence-assisted clustering technique has been applied to identify the main phases of magnetic alloys [12]. In addition, a machine learning-based approach for the classification of crystal systems and space groups was introduced using tools such as K-Nearest Neighbors and extremely randomized trees [13]. On the other hand, to the knowledge of the authors, the XRD line profiles have not been evaluated by machine learning yet; therefore, this is the first artificial intelligence-assisted XLPA procedure in the literature.

## 2. Experimental Material and Methods

### 2.1. Processing of the Co-Cr-Fe-Ni CCA Film

Co-Cr-Fe-Ni CCA combinatorial thin film was manufactured by a recently developed MBS device (manufacturer: Polygon Physics, Fontaine, France). This technique uses multiple independent ion sources in a source ring around the target holder. The wafer is positioned above the target holder. The orientation and number of ion sources make the premanufactured CCA target redundant; rather, in this technique, single element targets are used for ion sputtering, and due to an appropriate positioning of the targets, the required combinatorial sample with a well-defined element gradient could be formed on the surface of the substrate. Altogether, twelve independent, commercially pure Co, Cr, Fe and Ni targets were arranged around the circular Si single crystal substrate, which had a diameter of 10 cm. For each element, three targets were placed next to each other. More details of the MBS technique and the specific parameters of deposition have been published in Ref [9].

### 2.2. Measurement of the X-ray Diffraction Patterns Using Synchrotron Radiation

The microstructure of the combinatorial Co-Cr-Fe-Ni CCA sample was characterized by XLPA. The X-ray diffraction patterns were measured using synchrotron radiation at the Deutsches Elektronen-Synchrotron (DESY) in Hamburg, Germany. The wavelength of X-rays was 0.028178 nm since the beam energy was selected as 44 keV. The Co-Cr-Fe-Ni CCA disk was investigated in approximately 1000 positions arranged in a matrix containing 25 horizontal and 44 vertical coordinates. The spacing between the measured locations was 4040 μm horizontally and 2000 μm vertically. The positions of the studied sites on the sample surface were described by the coordinates *y* and *z*, which varied in the ranges from 8 mm to 94 mm and from −2.4 mm to 4.1 mm, respectively. An angle of 3.84° was set between the incident beam and the sample surface. Therefore, although the beam size was only 200 µm horizontally and 1000 µm vertically, the horizontal footprint of the beam was larger (about 1500 µm) due to the low inclination angle. A Varex 4343 detector was used for recording the diffraction patterns with a sample–detector distance of 1 m. The acquisition time for each pattern was 5 s. Due to the small illuminated volume, six patterns were recorded at each measurement position which were summed up and integrated in order to obtain a diffractogram for evaluation. Further details of the XRD measurement were published in a previous paper [11].

The XRD patterns were evaluated for the microstructure using the novel ML-XLPA method. The details of this new technique will be presented in the next section since the development of this novel method is the main result of this paper. For validating the new ML-XLPA procedure, some patterns were also evaluated with the traditional convolutional multiple whole profile (CMWP) fitting technique [14]. In this method, the experimental pattern is fitted with a calculated one in which each peak is determined as the convolution of the theoretical intensity profiles related to the crystallite size distribution, and the distortion caused by dislocations and planar defects. These calculated peaks are added to a background spline yielding the pattern used for fitting. The parameters of the microstructure are determined from best agreement between the measured and calculated patterns. For FCC structures, the three most important microstructural parameters obtained from the CMWP method are the area-weighted mean crystallite size, the density of dislocations and the twin fault probability. Additional information about the CMWP procedure can be found in [14].

## 3. Development of the ML-Based XLPA Methodology

### 3.1. Steps of the ML-XLPA Method

**Figure 1:**
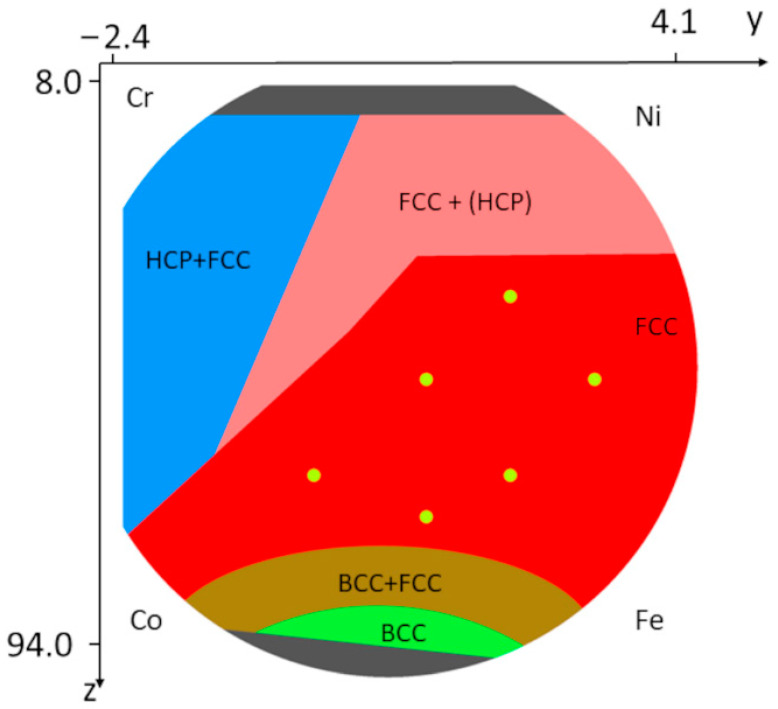
Phase map of the studied Co-Cr-Fe-Ni CCA film. The six small spots indicate locations where the results of the new ML-XLPA method are compared with the microstructural parameters obtained by the traditional CMWP full pattern fitting.

**Figure 2:**
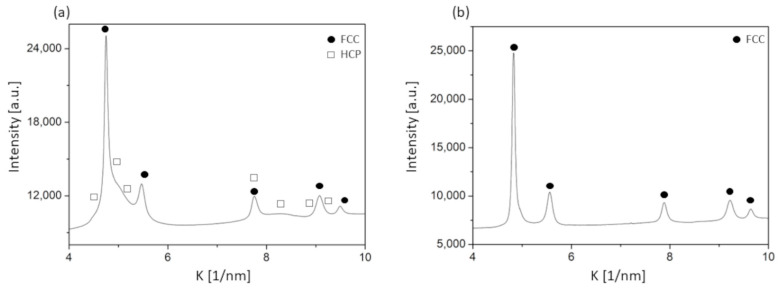
Illustrative examples for the XRD patterns taken on selected points in the (**a**) FCC+(HCP) and (**b**) FCC regions of the Co-Cr-Fe-Ni CCA film.

In our previous works [11,15], a phase map of the presently studied Co-Cr-Fe-Ni CCA sample was determined, which is also presented here in Figure 1. HCP and BCC indicate hexagonal close-packed and body-centered cubic structures, respectively. In the region marked by FCC+(HCP), the existence of a minor HCP phase is only guessed from the broad peaks that appeared on the background or the shoulders of the FCC peaks in the diffractograms. As an example, Figure 2a shows an XRD pattern from the FCC+(HCP) region. It should be noted that, in our former publications, this region was a part of the single phase FCC area, since the broad peaks were included in the background for the points where CMWP evaluation was performed [11,15]. In the present case, the FCC and FCC+(HCP) regions are separated since the new ML-XLPA method was developed only for single phase FCC structures. As an example Figure 2b shows an XRD pattern from the FCC region. This distinction was necessary because the ML-XLPA is sensitive to the severe asymmetry caused by minor secondary phases. For the single phase FCC diffractograms, the background is very smooth, therefore the ML-XLPA method can recognize and evaluate the peaks unambiguously. The ML-XLPA utilizes a learning set that was created using theoretical diffraction profiles. Therefore, the significant asymmetric broadening, shown in Figure 2a, at the right side of the first FCC peak could not be handled currently.

Since the FCC area is the largest single phase region in the studied CCA film (see Figure 1), the ML-XLPA procedure was first developed for this structure. The single phase FCC region contains more than 300 measurement points, i.e., the diffractograms obtained in these points should be evaluated. The evaluation of such a vast amount of data with traditional methods, such as CMWP pattern fitting, would consume an unreasonably long time. Therefore, increasing the efficiency of evaluation is highly motivated.

**Figure 3:**
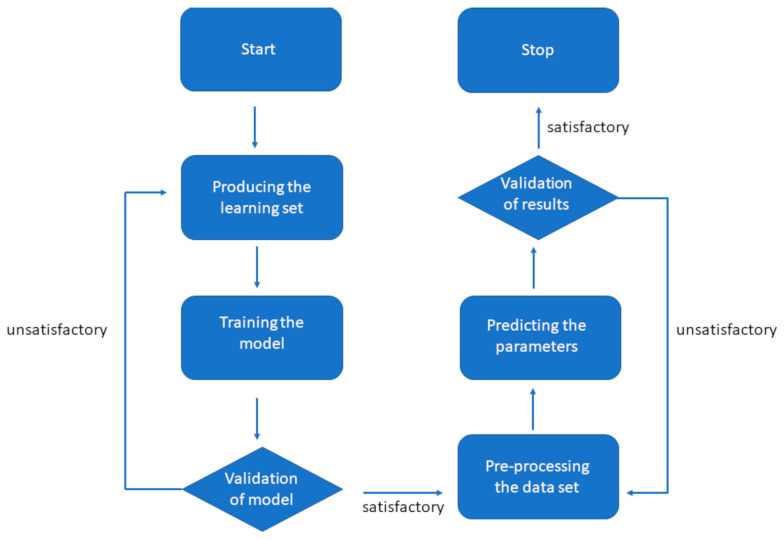
Flowchart of the ML-XLPA procedure.

The ML-XLPA method contains four main steps: the production of the learning set (c.f. next subsection), the training of the model, the preprocessing of the data, and the prediction of the characteristic structural parameters from the diffractograms. The learning set is produced by calculating the peak profiles from the parameters of the microstructure. The intensity and position of the peaks are perturbed around a fixed value. The peak positions were calculated from the lattice constant of the FCC phase determined in our former studies. The value of the lattice constant remained unchanged within the experimental error in the FCC region (0.360 ± 0.001 nm), as revealed in our former publication [11]. Gradient boosting was chosen as the machine learning method from the XGBoost library to predict the characteristic parameters of the nanostructure [16]. The measured diffractograms were preprocessed by subtracting the background with the ARPLS method [17]. Both the learning set and the final prediction were individually validated. The flow chart of the process is shown in Figure 3.

### 3.2. Production of the Theoretical XRD Patterns Used as the Learning Set

First, a learning XRD pattern set was calculated using well-defined theoretical functions of the diffraction peaks which depend on the parameters of the microstructure. In the calculation presented below, the scattered intensity at diffraction vector *K* is denoted by *I*(*K*). The XRD pattern is mainly influenced by the dislocation density ρ, the twin fault probability *β*, and the median and the log-normal variance, *m* and $\sigma^{2}$, respectively, of the crystallite size distribution, which together determine the average crystallite size. In this work, the area-weighted mean crystallite size (${\langle x\rangle }_{area}$) was calculated, given by the following equation:

(1) $${\langle x\rangle }_{area}=m\cdot exp\left(2.5\sigma^{2}\right)$$

We propose a supervised learning method to infer the mapping

(2) $$I\left(K\right)\mapsto \left(\begin{array}{c} \begin{array}{c} \beta \\ \rho \end{array}\\ \begin{array}{c} m\\ \sigma \end{array} \end{array}\right).$$

This, however, requires a considerable amount of labeled training data. Since the inverse mapping, the one returning the XRD pattern as a function of ρ, β, σ and m, is known from theory [18], we sample this four dimensional parameter space and generate XRD patterns to end up with around 10^5^ labeled patterns. The parameters are sampled uniformly from ρ∈[0.005;0.05] in nm^−2^, β∈[0;5] in percentage, m∈[10;30] in nanometer. The parameter σ is sampled log-uniformly from the interval [0.01; 0.8].

**Figure 4:**
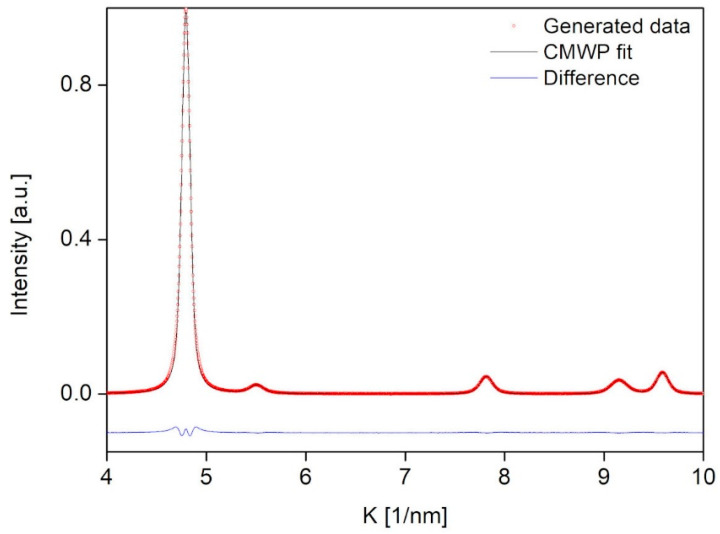
An FCC XRD pattern obtained by the method used for the production of the learning set (indicated by red circles). For validating the correct operation of the method, the calculated pattern was fitted by the CMWP method, which is illustrated by the black line in this figure. At the bottom, the difference between the calculated and the fitted patterns is also shown by blue line.

The intensity of a single peak, *I*(*K*), was obtained as the convolution of the theoretical intensity functions from different contributions. These contributions depend on the structural parameters. If we assume that the XRD peak broadening is caused by the crystallite size, dislocations and twin faults, the intensity of a single peak can be calculated as:

(3) $$I\left(K\right)=I{\left(K\right)}_{S}*I{\left(K\right)}_{D}*I{\left(K\right)}_{F}.$$

where $I{\left(K\right)}_{S}$, $I{\left(K\right)}_{D}$ and $I{\left(K\right)}_{F}$ are the intensity peak profiles related to the crystallite size, dislocations and twin faults, respectively. The symbol ∗ represents convolution. For each peak, the individual contributions were evaluated in the Fourier-space, i.e., the Fourier transforms of the theoretical intensity profiles were calculated. Then, their product was inverse Fourier transformed by fast Fourier transform. The final XRD pattern was obtained as the sum of the peaks, i.e.,

(4) $$I\left(K;\rho ,m,\sigma ,\beta \right)=\sum_{j=1}^{N_{p}}\alpha_{j}I_{j}\left(K-K_{j}^{\left(0\right)};\rho ,\beta ,m,\sigma \right),$$

where $K_{j}^{\left(0\right)}$ is the position of the *j*th peak and $\alpha_{j}$ is its intensity. Further microstructural parameters such as the effective cut-off radius of dislocations (R_e_^*^) and the two quantities *q* and C_h00_ describing the average contrast factor ($\bar{C_{hkl}}$) were fixed to specific values since these parmeters do not change significantly in the different locations investigated by the CMWP method in our former works [11,15]. Thus, in the present study, the values of R_e_^*^, C_h00,_ and *q* were fixed to 8 nm, 0.31, and 2.1, respectively. In the FCC region of the investigated sample, the value of the Burgers vector was 0.255 nm, which was obtained by dividing the lattice constant 0.36 nm with square-root of two. One diffractogram comprises the sum of five individual peaks (*N_p_* = 5). These are the 111, 200, 220, 311 and 222 peaks of the FCC phase. The intensities and the peak positions chosen are fixed values consistent with the previous analysis of the sample. To make the training set more general, we allow the intensity and the peak position to vary randomly by a relative magnitude of 2% for each peak. In addition, we add Gaussian white noise to the XRD pattern with a relative magnitude of 0.01%. The learning set was validated by generating a diffractogram via the introduced method with the following parameters: area-weighted mean crystallite size: 13.2 nm, dislocation density: 200 × 10^14^ m^−2^, and twin fault probability: 3%. A CMWP fit was carried out on the calculated pattern to estimate the microstructural parameters. The generated and the CMWP fitted diffractograms, as well as the difference between them, are plotted in Figure 4. For the area-weighted mean crystallite size, CMWP gave 12.8 nm, while for the dislocation density and the twin fault probability we obtained 202 × 10^14^ m^−2^ and 3.1%, respectively. The parameters received by CMWP fitting agreed well within the error with the initial parameters of the generated diffractogram. Therefore, the learning set could be accepted as a satisfactory dataset of labeled diffractograms.

**Figure 5:**
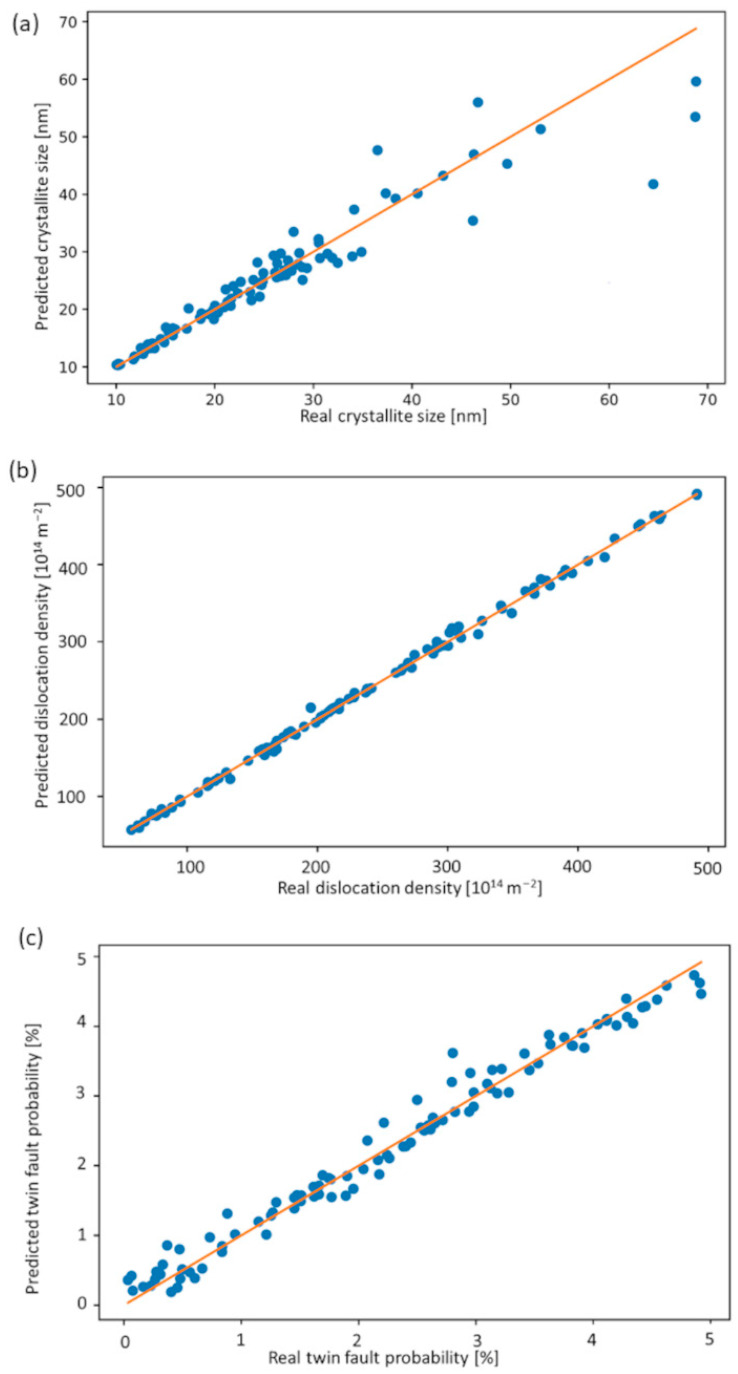
The real values of the crystallite size (**a**), dislocation density (**b**) and twin fault probability (**c**) versus the predicted ones obtained by ML-XLPA for the control set of diffractograms.

Using the as-prepared learning set, a model teaching was performed as the next step of ML-XLPA. We solve the regression problem with gradient boosting implemented in the XGBoost library. A regular winner in ML competitions, “eXtreme Gradient Boosting” or XGBoost is a highly scalable and computationally efficient implementation of gradient boosting trees. Boosted decision trees are a sequentially added ensemble of decision trees. Each additional tree is trained to correct errors made by previous trees until no further corrections can be made on a validation dataset. Gradient boosting grows the best trees by optimizing a loss function consisting of the prediction error and a regularization term describing the complexity of the trees. Depending on the loss function, XGBoost can be run in both classification and regression modes to predict categorical or continuous values [16,19]. Although there is an indication that a neural network-based model could be used especially for classification [20], we demonstrate that even classical machine learning methods can reliably identify the structural parameters of a given XRD pattern. On top of the built in regularization of XGBoost, to avoid any possible overfitting, we applied a 90–10 cross-validation during the training process and applied an early stopping condition. We have evaluated the performance of the model on an independent test set. In Figure 5, the predicted values of the parameters are plotted against the actual parameters of the generated dataset used for validation. The error is estimated as the standard deviation of the difference of the predicted and the actual parameters. The absolute error of the parameters in the case of the crystallite size, dislocation density, and twin fault probability was 3.76 nm, 4 × 10^14^ m^−2,^ and 0.2%, respectively. The low values of absolute errors show that in the case of single phase, artifact-free diffractograms, the prediction is highly accurate.

### 3.3. Mapping of the FCC Microstructure of the Combinatorial Co-Cr-Fe-Ni CCA Film Using the ML-XLPA Method

**Figure 6:**
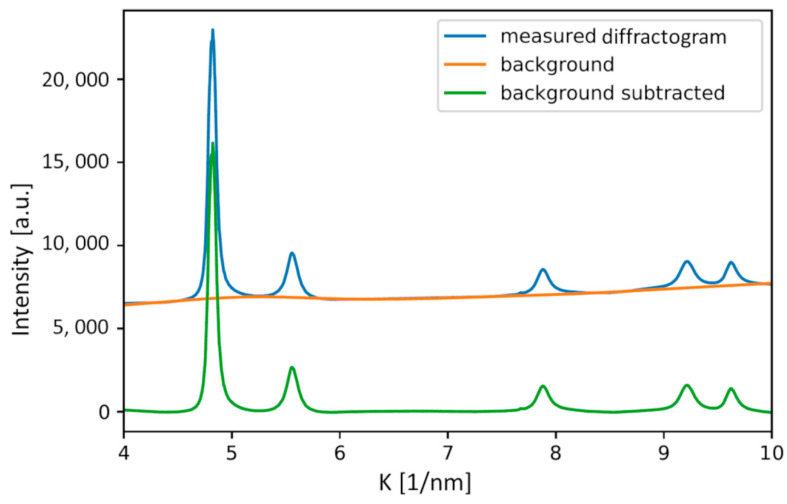
Illustration of the background subtraction for a measured FCC diffraction pattern.

As previously mentioned, weak secondary phase peaks and other artifacts have a significant influence on the quality of the prediction. Therefore, the preprocessing has a severe impact on the precision of the predicted parameters. In the case of the dataset used in the present work, the background of the diffractograms varied significantly. The algorithm for background correction relies on iterative smoothing, referred to as asymmetrically reweighted penalized least squares (ARPLS). For the numerical implementation, we used the Pybaselines library. Signal processing of data from experimental techniques, such as Raman, FTIR, NMR, XRD, XRF, PIXE, etc., is often hindered by the drift of baselines. This may often blur signals and deteriorate final extracted results. Polynomial fitting is frequently used for correction but it cannot be applied automatically and often requires manual intervention, especially in low SNR cases. The ARPLS algorithm, implemented as a Python library Pybaselines, provides a number of different algorithms for performing an automated baseline correction. The program’s interface makes possible rapid testing and comparison of multiple baseline correction algorithms to find the best one for the given dataset [17,21]. Figure 6 shows an illustrative example for a typical experimental diffractogram along with its background and the diffractogram after background subtraction. The latter pattern was evaluated by the new ML-XLPA method.

The running time of the whole procedure is on the order of hours. Most of this time is taken by the production of the learning set and the training of the model. The determination of the microstructural parameters for the studied area of the sample (the last step) required only some seconds.

**Figure 7:**
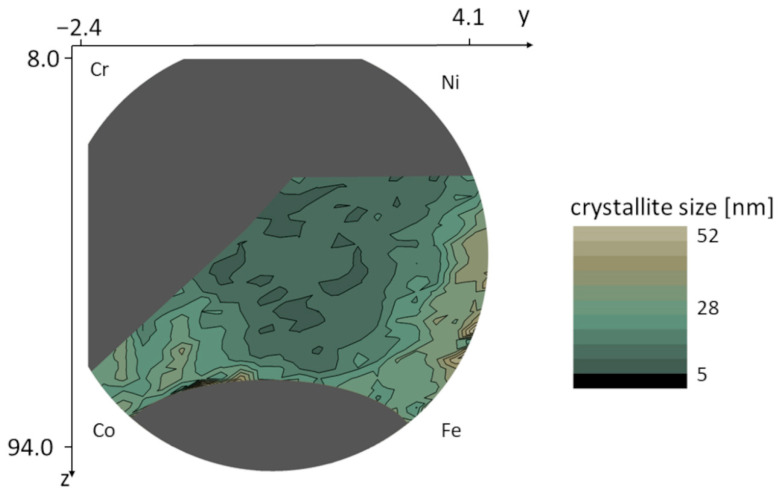
Variation of the crystallite size in the FCC region of the combinatorial Co-Cr-Fe-Ni CCA film.

**Figure 8:**
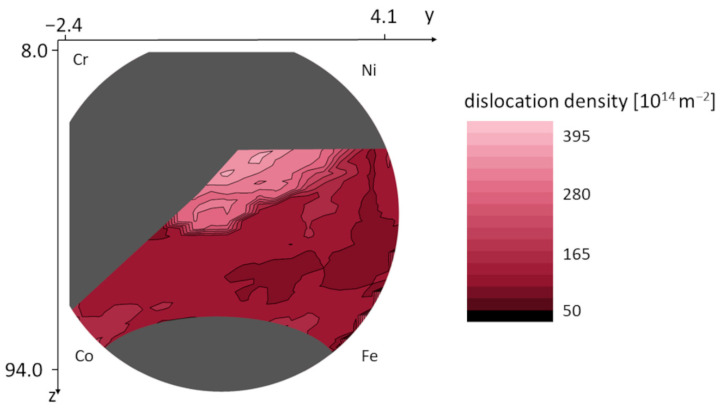
Variation of the dislocation density in the FCC region of the combinatorial Co-Cr-Fe-Ni CCA film.

**Figure 9:**
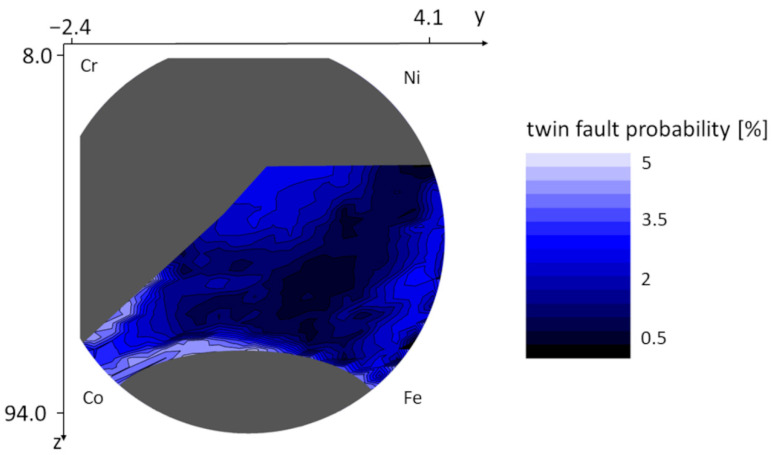
Variation of the twin fault probability in the FCC region of the combinatorial Co-Cr-Fe-Ni CCA film.

The values of the microstructural parameters predicted by ML-XLPA method were plotted as color maps over the silhouette of the wafer. The grey areas represent the locations where the diffractograms were not suitable for prediction due to different main phases or the presence of noticeable secondary phases. In the case of the area-weighted mean crystallite size, the range of the observed values was between 9 and 36 nm, as shown in Figure 7. Figure 8 shows the dislocation density map. The values on the map are between 85 and 460 × 10^14^ m^−2^. The map of the twin fault probability is shown in Figure 9. The values vary between 0.6 and 4.2%.

## 4. Discussion

### 4.1. Comparison of the Microstructural Parameters Obtained from the ML-XLPA Method and CMWP Pattern Fitting

**Figure 10:**
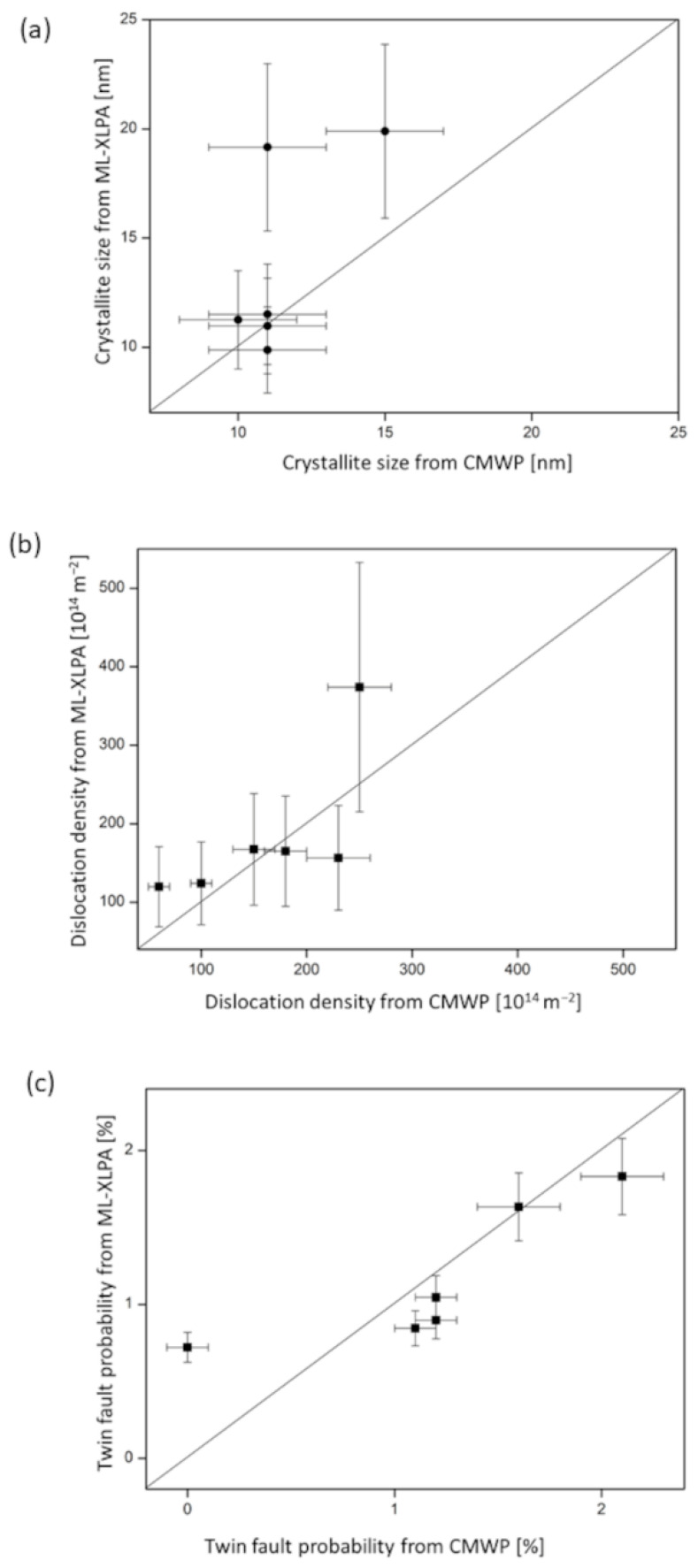
The crystallite size (**a**), dislocation density (**b**) and twin fault probability (**c**) obtained by ML-XLPA versus the values determined by the CMWP fitting method at the six points shown in Figure 1.

The parameter maps obtained by ML-XLPA were compared in six positions to the parameters acquired through CMWP pattern fitting. These positions are shown in Figure 1. The microstructural parameters obtained in these points using CMWP have already been listed in our former publication [15]. The error of the predicted parameters was estimated from the difference between the predicted values obtained for different positions where CMWP yielded identical or near-identical parameter values. The relative error of the area-weighted mean crystallite size, the dislocation density and the twin fault probability were 20%, 43% and 14%, respectively. The validation diagrams for the area-weighted mean crystallite size, the dislocation density and the twin fault probability are shown in Figure 10a–c, respectively. The values of ML-XLPA and CMWP are adequately close, although the error of the estimate is high in the case of the dislocation density. This high error can be attributed to the sensitivity of the dislocation density value to the parameter setting in the ARPLS method applied for background determination. Conversely, a slight change in the ARPLS parameters may result in a significant variation of the tail part of the peaks after the background subtraction, which then strongly influences the value of the dislocation density. The background is especially uncertain at the peaks appearing at high diffraction angles due to their strong overlapping (between K = 9 and 10 nm^−1^). The uncertainty in the background determination at high diffraction angles mainly influences the dislocation density since the contribution of dislocations to peak broadening increases with increasing K. In spite of the elevated error of the dislocation density, the trends observed in the dislocation density map are expected to be reliable since the same parameters were applied in the background subtraction.

### 4.2. Variation of the Microstructure in the FCC Phase Region of the Combinatorial Co-Cr-Fe-Ni CCA Film

Figure 7, Figure 8 and Figure 9 show that in the Co-Cr-Fe-Ni system, the parameters of the microstructure change with the chemical composition. Namely, the crystallite size, the dislocation density and the twin fault probability varied in the ranges 9–36 nm, 85–460 × 10^14^ m^−2^ and 0.6–4.2%, respectively. The lowest crystallite size (8–12 nm) was observed in the middle of the disk where each element’s concentration is at least 10%, i.e., the alloy has four major components. The largest dislocation density of 300–400 × 10^14^ m^−2^ was obtained in the upper part of the FCC region with the coordinates of about *y* = 0.5 and *z* = 37 which corresponds to the chemical composition of about 30% Co–40% Cr–10% Fe–20% Ni (at.%). In this region, the twin fault probability is also high (about 4%). It should be noted, however, that the microstructural parameters change very smoothly, i.e., a relatively large region in the maps shows similar parameter values. This means that the microstructure in the Co-Cr-Fe-Ni system is not very sensitive to the chemical composition, at least for single phase FCC structures.

As a further development direction for the novel ML-XLPA method, we plan to extend the capability of this technique to the regions where the material is not a single phase FCC structure. This work implies (i) handling of non-smooth background including weak peaks of secondary phase(s) (see Section 3.1), (ii) teaching the program for evaluation of BCC and HCP structures and (iii) making ML-XLPA suitable for the determination of the microstructural parameters when multiple phases with similar fractions co-exist. Also, by improving the pre-processing procedure, the error of the dislocation density should be mitigated. We also plan to make the software freely available for any user.

## 5. Conclusions

1. A novel characterization method for FCC nanostructures was developed in which machine learning is combined with the analysis of X-ray diffraction peak profiles. It was demonstrated that the ML-XLPA technique is able to evaluate a large number of XRD patterns in a short time, yielding maps of the microstructural parameters, such as the crystallite size, the dislocation density, and the twin fault probability.

2. The applicability of the new ML-XLPA method was illustrated on a combinatorial Co-Cr-Fe-Ni CCA film manufactured by PVD. Microstructural mapping with a reasonable resolution and in a reasonable time can be performed only if both the data acquisition and the evaluation are fast enough. Therefore, in the present case, the XRD patterns were measured at a synchrotron and were then evaluated by the novel ML-XLPA method. It was found that in the FCC region of the combinatorial Co-Cr-Fe-Ni CCA film, the crystallite size varied between 9 and 36 nm. The dislocation density and the twin fault probability were in the ranges of 85–460 10^14^ m^−2^ and 0.6–4.2%, respectively.

3. The new ML-XLPA method was elaborated only for single phase FCC materials, and further development is required for its extension to other structures, such as HCP or BCC. The improvement of ML-XLPA for the characterization of other single phase structures is relatively straightforward, since it requires only the production of a learning set of a large number of XRD patterns using the program developed in this study. Much more work is needed to extend this method to multiphase materials, especially when the diffraction peaks of the different phases strongly overlap.

## Data Availability

The measured and the evaluated data of this study are available on request from the corresponding author.
